# A Wideband Millimeter-Wave Dual-Beam Dielectric Resonator Antenna with Substrate Integration Capability

**DOI:** 10.3390/mi15081022

**Published:** 2024-08-10

**Authors:** Jin Shi, Ranhao Xu, Bowen Wu, Lei Wang, Ruirui Jiang

**Affiliations:** 1 School of Information Science and Technology, Nantong University, Nantong 226019, China; jinshi0601@hotmail.com (J.S.); xuranhao2030@outlook.com (R.X.); bowenwu1998@outlook.com (B.W.); 2 Research Center for Intelligent Information Technology, Nantong University, Nantong 226019, China; 3 Nantong Key Laboratory of Advanced Microwave Technology, Nantong University, Nantong 226019, China; 4 College of Information Engineering, Yancheng Institute of Technology, Yancheng 224051, China; wanglei0324@ycit.edu.cn

**Keywords:** dielectric resonator antenna (DRA), dual-beam antenna, millimeter-wave, substrate integrated, wideband

## Abstract

A wideband dual-beam dielectric resonator antenna (DRA) with substrate integration capability was proposed for millimeter-wave (mm-wave) applications. The four rows of air vias along the *x*-direction and two extended rectangular patches could shift the undesirable radiation mode upward and move the conical-beam radiation mode downward, respectively. Thus, the TE_211_ mode and the TE_411_ mode of the patch-loaded perforated rectangular substrate integrated dielectric resonator (SIDR) supporting the dual-beam radiation can be retained in the operating band, and their radiation can be improved by the air vias along the *y*-direction. The T-shaped line coupled dual-slot structure could excite the above two modes, and a dual-slot mode supporting dual-beam radiation could also work. Then, a wideband DRA with a stable dual-beam radiation angle can be achieved, and its impedance matching can be improved by two air slots on two sides. Compared with the state-of-the-art dual-beam antennas, the proposed antenna shows a wider bandwidth, a higher radiation efficiency, and the substrate integration capability of DRA, making it more suitable for mm-wave applications. For demonstration, a 1 × 4 array was designed with the 10 dB impedance matching bandwidth of 41.2% and the directions of the dual beams between ±30° and ±35°.

## 1. Introduction

With the growth in mobile users leading to channel congestion, dual-beam antenna could offer increased channel capacity compared to single-beam antenna or omnidirectional antenna [1], addressing the issue of data traffic problems. Using multiple antenna elements is an effective way to achieve dual-beam radiation, such as the beamforming network with multiple antenna elements [2,3,4], the lens antenna or the planar metasurface antenna with two excitation sources [5,6,7,8], the composite right-/left-handed structure in a leaky-wave antenna [9,10,11], and combining multiple gradient refractive index metamaterial unit cells with a slot antenna [12]. All of these could achieve dual-beam radiation with large gains, but their excessively large size or complex non-planar structures restrict their application to some extent. Thus, it is necessary to explore dual-beam radiation in a single radiator to reduce the size and complexity. 

Currently, most dual-beam antennas in a single radiator are patch antennas [13,14,15,16,17]. The TM_02_ mode in a patch antenna can support dual-beam radiation, but the bandwidth is limited if only the TM_02_ mode works. To extend the bandwidth of the dual-beam patch antenna, an additional reflective zero was provided by etching the U-slots [13,14] and L-shaped slots [15] on the patch or utilizing a cross-shaped feeding probe to excite the patch [1]. As a result, the bandwidth of the dual-beam patch antenna can be broadened to 15%. In [16], a pair of T-shaped metallic strips could move the TM_22_ mode of the patch close to the TM_02_ mode of the patch, forming a bandwidth of 19.4%. In [17], the inserted strip under the patch made the antenna have two TM_02_ modes to provide dual-beam radiation, and the bandwidth could be extended to 23.5%. However, the bandwidth of the dual-beam antenna requires further improvement from the perspective of wideband operation. Furthermore, the conductive loss of a millimeter-wave (mm-wave) patch antenna would reduce the radiation efficiency to some extent. 

Utilizing a dielectric resonator antenna (DRA) element to achieve dual-beam radiation [18,19] could exhibit the advantages of high radiation efficiency and large design flexibility. In [18], switchable parasitic metal robs were utilized as directors around an omnidirectional DRA to achieve dual-beam radiation. In [19], a pair of face-to-face triangular DRAs with top metal surfaces could achieve dual-beam radiation, where each DRA contributed one radiation beam. However, the non-planar radiator and parasitic structures made it hard to apply in mm-wave applications with a high integration level. Substrate integrated DRAs (SIDRAs) [20,21,22,23,24] can greatly enhance the integration level of mm-wave DRA, simplifying the manufacturing process and reducing assembly error while also maintaining high radiation efficiency. Thus, the SIDRA is preferred for mm-wave dual-beam antenna. 

In this paper, the TE_211_ mode and the TE_411_ mode of the substrate integrated dielectric resonator (SIDR), as well as the dual-slot mode, all support dual-beam radiation. They can form a wide bandwidth of 41.2% with a stable dual-beam radiation angle by moving two undesirable modes outside of the operating band. Meanwhile, all the structures of the antenna are planar and suitable for the PCB process, so the proposed antenna has substrate integration capability. Thus, wideband dual-beam radiation, high integration level, and high radiation efficiency can all be achieved, which make it preferable to mm-wave applications. The operating mode, impedance matching, mode control, and radiation characteristics of the proposed antenna are analyzed in detail. A 1 × 4 array was constructed to validate the above analysis. Compared with the state-of-the-art designs, this design highlights its superiority in terms of bandwidth, radiation efficiency, dual-beam characteristic, and integration. 

## 2. Design of the Proposed Antenna Element

### 2.1. Configuration of the Proposed Antenna Element

**Figure 1:**
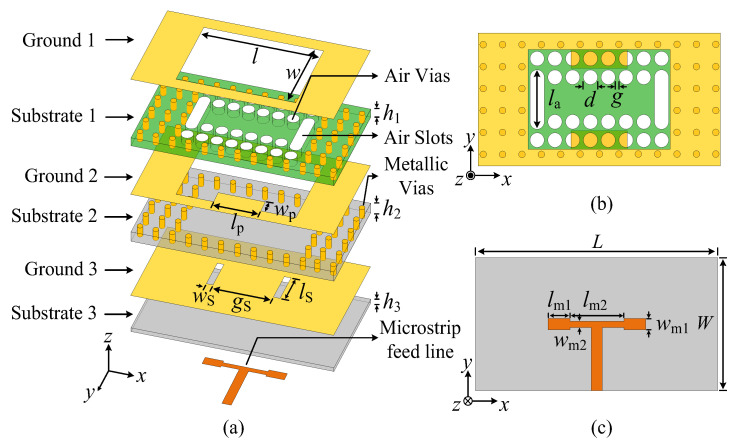
Configuration of the proposed dual-beam substrate integrated dielectric resonator antenna (SIDRA) element. (**a**) Exploded view. (**b**) Top view. (**c**) Bottom view.

The configuration of the proposed wideband dual-beam SIDRA is shown in Figure 1, which consists of a patch-loaded perforated rectangular SIDR, a T-shaped line, and two parallel coupling slots. In the patch-loaded perforated rectangular SIDR, four rows of air vias along the *x*-direction and two air slots along the *y*-direction are drilled in the substrate with high permittivity (Substrate 1), and a pair of rectangular patches are symmetrically extended from the longer edges of the middle ground layer (Ground 2). The substrates of the proposed SIDR have two layers, which can offer greater design flexibility. The higher-permittivity one is RT6010 substrate with a permittivity (*ε_r_*_1_) of 10.2, which is beneficial to maintain the SIDR resonant condition and reduce the size of the radiator, while the lower-permittivity one is RO4003C substrate with a permittivity (*ε_r_*_2_) of 3.38, which is beneficial to reduce the Q-factor to support wideband operation when combined with the dielectric resonator (DR) [25]. Furthermore, peripheral metallic vias with three metallic layers are placed around the DR as the metallic cavity. 

The T-shaped microstrip line, containing Substrate 3 with a permittivity (*ε_r_*_2_) of 3.38, is the feed circuit of the proposed antenna and symmetrically excites the two parallel coupling slots on the bottom ground layer (Ground 3). The feedline adopts a stepped impedance configuration considering the impedance transformation in power division. The full-wave simulation is performed by using Computer Simulation Technology (CST) Microwave Studio 2017. 

### 2.2. Operating Mechanism of the Antenna Element

**Figure 2:**
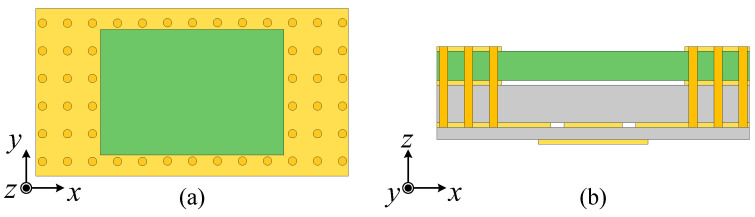
Configuration of Ant. I. (**a**) Top view. (**b**) Side view.

**Figure 3:**
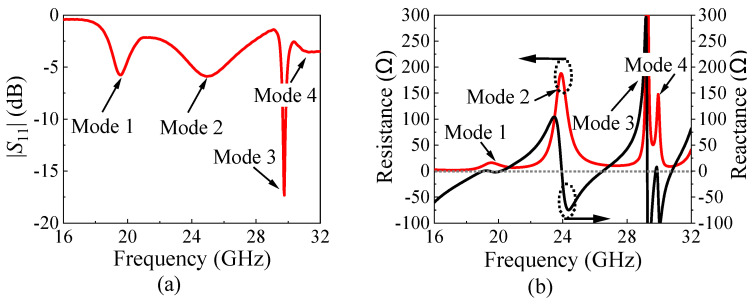
Simulated |*S*_11_| and the input impedance of Ant. I. (**a**) |*S*_11_|. (**b**) Input impedance (red line: the real part; black line: the imaginary part).

**Figure 4:**
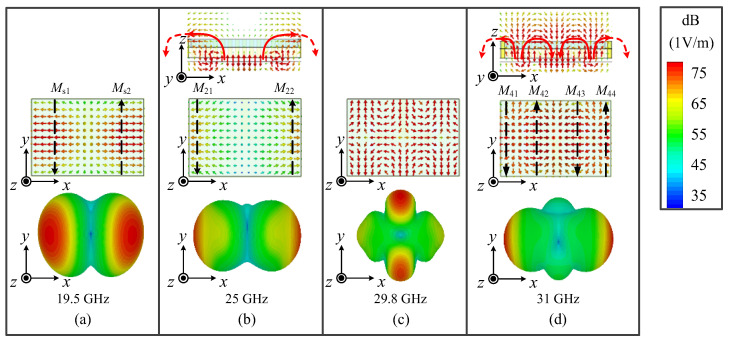
The simulated results of *E*-fields and 3-D radiation patterns of Ant. I for (**a**) mode 1 (the dual-slot mode), (**b**) mode 2 (the TE_211_ mode), (**c**) mode 3, and (**d**) mode 4 (the TE_411_ mode).

In order to clarify the operating mechanism of the proposed antenna, Ant. I with a basic SIDR is given first, as shown in Figure 2, where the basic SIDR just includes stacked substrates and the metallic cavity and is excited by the T-shaped line coupled dual slots. The size of the air gap between Substrates 1 and 2 is 0.034 mm. Ant. I could achieve four resonant points in the operating band, as indicated by the |*S*_11_| curve in Figure 3a and the input impedance curves in Figure 3b. The four resonant points correspond to mode 1, mode 2, mode 3, and mode 4, respectively. The electric fields (*E*-fields) of the four modes are shown in Figure 4.

It was found that mode 1 is a dual-slot mode excited by the T-shaped line, which is equivalent to two antiphase magnetic currents (MCs, *M*_s1_ and *M*_s2_), providing symmetrically reversed *E*-fields inside the SIDR. Thus, dual-beam radiation pointing to two oblique directions can be observed [26], as shown in Figure 4a. 

Mode 2 is the TE_211_ mode of the basic SIDR and can be excited by the dual slots. Its *E*_x_ performs only a half variation of the field inside the SIDR due to the equivalent electric wall of the metallic vias, and two half variations of the virtual field could be compensated, considering the mirror effect as shown in Figure 4b. Thus, mode 2 is also equivalent to two antiphase MCs (*M*_21_ and *M*_22_). The radiation pattern is similar to a bidirectional radiation and shows a large difference to that of mode 1 because the MCs and *E*_x_ are concentrated on left and right ends of the SIDR. 

The *E*-field distribution and 3-D radiation patterns of mode 3, which is an unwanted mode, are shown in Figure 4c. In this mode, the strong reversed *E*_y_ is inside the basic SIDR, which produces the dual-beam radiation along the *y*-direction, as shown in Figure 4c. This radiation pattern differs from the desired dual-beam radiation pattern along the *x*-direction. Therefore, this mode is an undesirable one, and it should be removed outside of the operating band to ensure the stability of radiation within the operating band. 

Mode 4 is the TE_411_ mode of the basic SIDR, which has two complete and two half variations of the field, and two half variations of the virtual field could also be compensated. Thus, mode 4 can be seen as four equivalent MCs (*M*_41_, *M*_42_, *M*_43_, and *M*_44_) to support dual-beam radiation. However, the strong *E*_z_ makes the beam elevation angles too low, acting like bidirectional radiation, as shown in Figure 4d.

In summary, Ant. I achieves dual-beam radiation for the dual-slot mode, but it has some problems to be addressed, such as removing the unwanted mode (mode 3), the radiation direction of mode 2 and mode 4, and the poor impedance matching. Despite this, the resonant frequencies of the modes of SIDR (the TE_211_ mode and the TE_411_ mode) can be expressed in the formula. Considering the equivalent electric wall formed by the metallic vias, the number of half waves along the *x*-direction should subtract one because the one-half wave near the two edges of SIDR is reduced to one-quarter wave [27], so that the resonant frequencies of the two modes can be calculated by

(1) $$f_{{\mathrm{TE}}_{211}}=\frac{c}{2\sqrt{\varepsilon_{reff}}}\sqrt{{\left(\frac{1}{l}\right)}^{2}+{\left(\frac{1}{w}\right)}^{2}+{\left(\frac{1}{2\left(h_{1}+h_{2}\right)}\right)}^{2}}$$

(2) $$f_{{\mathrm{TE}}_{411}}=\frac{c}{2\sqrt{\varepsilon_{reff}}}\sqrt{{\left(\frac{3}{l}\right)}^{2}+{\left(\frac{1}{w}\right)}^{2}+{\left(\frac{1}{2\left(h_{1}+h_{2}\right)}\right)}^{2}}$$,

respectively, where *l* and *w* are the length and width of the dielectric part, *h*_1_ is the height of Substrate 1, *h*_2_ is the height of Substrate 2, and *ε_reff_* is the effective relative dielectric constant of the basic SIDR. The effective dielectric constant *ε_reff_* can be written as

(3) $$\varepsilon_{reff}=\frac{\varepsilon_{r1}h_{1}+\varepsilon_{r2}h_{2}}{h_{1}+h_{2}}$$

where *ε_r_*_1_ is the relative dielectric constant of Substrate 1, and *ε_r_*_2_ is the relative dielectric constant of Substrate 2. The frequency of the dual-slot mode is determined by the length of the slots (*l*_s_).

**Figure 5:**
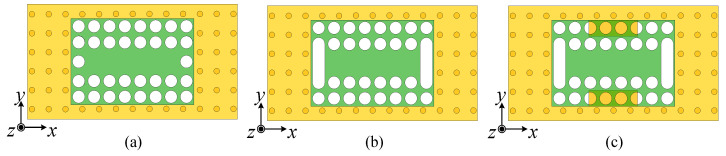
Top view of the reference antennas and the proposed antenna. (**a**) Ant. II. (**b**) Ant. III. (**c**) Proposed antenna.

**Figure 6:**
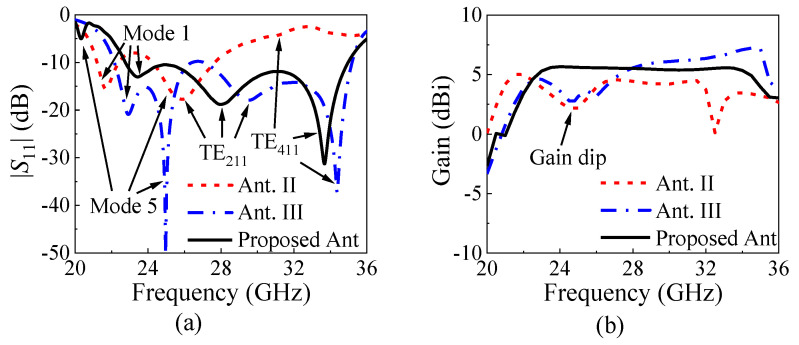
Simulated |*S*_11_| and the realized gain of the reference antennas and the proposed antenna. (**a**) |*S*_11_|. (**b**) Realized gain.

**Figure 7:**
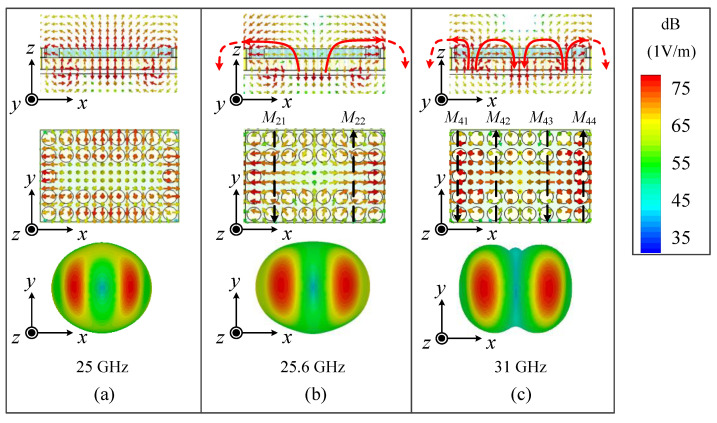
The simulated results of *E*-fields and 3-D radiation patterns of Ant. II for (**a**) mode 5, (**b**) mode 2 (the TE_211_ mode), and (**c**) mode 4 (the TE_411_ mode).

Ant. II adds four rows of air vias along the *x*-direction and two columns of air vias along the *y*-direction on Substrate 1 based on Ant. I, as shown in Figure 5a. The response of Ant. II is illustrated by the red curves in Figure 6. It can be seen that mode 1, the TE_211_ mode, and the TE_411_ mode still exist in the operating band, while the unwanted mode 3 has been removed because the air vias along the *x*-direction greatly affect its *E*_y_ components near the longer edges. However, it was found that there was a gain dip around 25 GHz, as shown in Figure 5b, corresponding to mode 5. The *E*-fields and radiation pattern of mode 5 are shown in Figure 7a, where *E*_x_ and *E*_y_ are concentrated near four edges, and strong *E*_z_ exists at the central area, resembling a cavity mode [28], resulting in conical-beam radiation and reduced gains in the dual-beam direction. Thus, mode 5 also needs to be removed outside of the operating band. 

On the other hand, the air vias along the *y*-direction in Ant. II make the distribution of *E*_x_ in mode 2 more uniform, which equivalently reduces the distance between two MCs, resulting in a raise in the beam elevation angles, as shown in Figure 7b. Meanwhile, the *E*_x_ component in mode 4 becomes the main *E*-field component both inside and above the SIDR, as shown in Figure 7c, which also raises the beam elevation angles so that their radiation patterns are similar to those of mode 1. However, the problems in Ant. II, such as the impedance matching and the gain dip, should be addressed.

**Figure 8:**
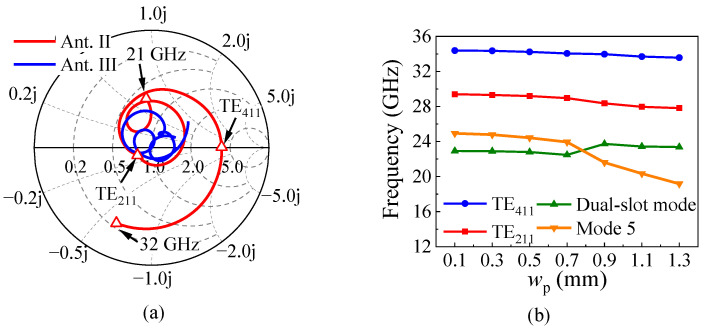
(**a**) The input impedances of Ant. II and Ant. III in the Smith chart. (**b**) The simulated frequency variation in different modes in *w*_p_.

Ant. III introduces a pair of air slots along the *y*-direction in Substrate 1 based on Ant. II, as shown in Figure 5b. From the blue curve of |*S*_11_| in Figure 6a, it is evident that Ant. III exhibits good impedance matching in the entire operating band. The four reflection zeros, corresponding to the dual-slot mode, mode 5, the TE_211_ mode, and the TE_411_ mode, are clearly visible. Figure 8a compares the input impedances of Ant. II and Ant. III in the Smith chart, finding that the curve of Ant. III is closer to the matching point and the impedance near the TE_411_ mode is the most improved. In additional, due to the reduced permittivity near the air slots, the TE_211_ mode moves faster than the TE_411_ mode, and they both move faster than the dual-slot mode, broadening the bandwidth of the antenna to some extent. However, the gain dip caused by mode 5 still exists near 25 GHz, as shown in the blue curve in Figure 6b. 

The proposed antenna extends a pair of rectangular patches from the longer edges of Ground 2 based on Ant. III, as shown in Figure 5c. Since mode 5 has strong *E*_y_ along the longer edges of the metallic cavity, the pair of patches affects mode 5 much more than the dual-slot mode, the TE_211_ mode, and the TE_411_ mode, as shown in the variation in Figure 8b, which suggests that mode 5 will move down outside of the operating band when *w*_p_ is large enough. This can be proven by the black curve in Figure 6a. 

**Figure 9:**
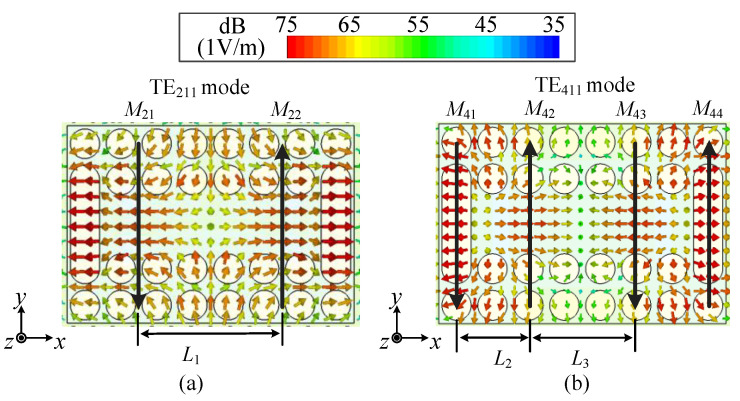
The simulated *E*-fields and the equivalent magnetic current models of the proposed antenna for (**a**) the TE_211_ mode and (**b**) the TE_411_ mode.

Figure 9 depicts the simulated *E*-fields and the equivalent magnetic current model of the proposed antenna for the TE_211_ mode and the TE_411_ mode. From the *E*-fields distribution in Figure 9, the TE_211_ mode and the TE_411_ mode can support stable symmetrical dual-beam radiation. The symmetrical dual-beam radiation characteristics of the TE_211_ mode and the TE_411_ mode in the proposed antenna can also be theoretically estimated. The TE_211_ mode can be viewed as a two-element magnetic current array, with two magnetic currents expressed as *M*_21_ and *M*_22_, separated by a distance of *L*_1_. The TE_411_ mode can be viewed as a four-element magnetic currents array, with four magnetic currents expressed as *M*_41_, *M*_42_, *M*_43_, and *M*_44_, while the distances between the two elements are expressed as *L*_2_ and *L*_3_. If considering that the far field radiated by magnetic current *M*_n_ can be expressed as *E*_n_, the two-element magnetic current array (the TE_211_ mode) can be expressed as

(4) $$E=E_{1}+E_{2}=E_{1}\left(1+\frac{M_{22}}{M_{21}}e^{jkL_{1}\sin\theta \sin\varphi }\right)$$

where *M*_21_ and *M*_22_ are equal in amplitude and opposite in phase. In the *E*-plane (*θ* = 90°), the far field can be deduced as

(5) $$F_{E}\left(\varphi \right)=E_{1\phi }\left(1+e^{j\left(kL_{1}\sin\varphi +\pi \right)}\right)$$

where *φ* represents the beam tilt angle. 

When extending the formula to the four-element magnetic current array (the TE_411_ mode), the far field in *E*-plane can be deduced as

(6) $$F_{E}\left(\varphi \right)=E_{3\phi }\left(1+a_{1}e^{j\left(kL_{2}\sin\varphi +\theta_{12}\right)}+a_{2}e^{j\left[k\left(L_{2}+L_{3}\right)\sin\varphi +\theta_{13}\right]}+a_{3}e^{j\left[k\left(2L_{2}+L_{3}\right)\sin\varphi +\theta_{14}\right]}\right)$$

where *a*_1_, *a*_2_, and *a*_3_ represent the amplitude ratios between *M*_42_ and *M*_41_, *M*_43_ and *M*_41_, and *M*_44_ and *M*_41_, respectively. *θ*_12_, *θ*_13_, and *θ*_14_ represent the phase differences between *M*_42_ and *M*_41_, *M*_43_ and *M*_41_, and *M*_44_ and *M*_41_, respectively. By using the above Formulas (4)–(6), it is possible to make a rough estimate of the *E*-plane radiation patterns when the antenna operates on the TE_211_ mode and the TE_411_ mode. 

In additional, the gain around the TE_411_ mode is slightly reduced due to the pair of patches. Thus, the proposed antenna obtains a flat gain inside the operating band, as shown in the black curve in Figure 6b. Finally, the three reflection zeros contribute a wide bandwidth of 42.2% with a stable dual-beam direction to the antenna.

### 2.3. Parametric Study on the Antenna Element

**Figure 10:**
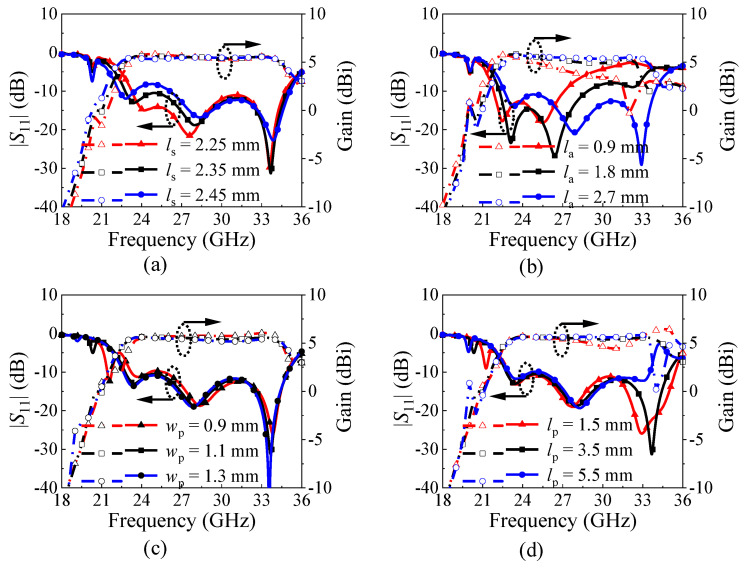
Simulated |*S*_11_| and the realized gain of the proposed dual-beam SIDRA for different values of (**a**) *l*_s_, (**b**) *l*_a_, (**c**) *w*_p_, and (**d**) *l*_p_.

Figure 10 shows the |*S*_11_| and gain variations of the proposed antenna with different *l*_s_, *l*_a_, *w*_p_, and *l*_p_. Figure 10a exhibits that the length (*l*_s_) of the dual-slot mainly affects the frequency of the first reflection zero. As *l*_s_ increases, the frequency of the first reflection zero decreases, while the TE_211_ mode and the TE_411_ mode just slightly move. This is in accordance with the variation trend of the dual-slot mode, which also confirms that mode 1 is the dual-slot mode. 

Figure 10b describes that the length (*l*_a_) of the air slots primarily affects the impedance matching for the upper half band of the proposed antenna. With the increase in *l*_a_, the impedance matching for the upper half band obviously improves, accompanied by a flattening realized gain curve. Meanwhile, the operating frequency of the antenna moves slightly upward with the increase in *l*_a_. When *l*_a_ is increased to 2.7 mm, the proposed antenna could achieve a good impedance matching and a flat gain curve within the operating band.

Figure 10c, d indicate how the width (*w*_p_) and length (*l*_p_) of the pair of patches affect the performance of the proposed antenna. It can be found from Figure 10c that the frequency of mode 5 decreases with the increase in *w*_p_, but too-large *w*_p_ will lead to a gain reduction within the operating band to some extent. Therefore, choosing an appropriate value of *w*_p_ allows mode 5 to be precisely shifted outside the band while maintaining good gain within the operating band. Figure 10d exhibits that *l*_p_ also affects the frequency of mode 5 to some extent, but it has a relatively small effect compared to *w*_p_. In addition, as *l*_p_ decreases, the upper edge of the antenna will shift upward to extend the bandwidth, but a gain dip occurs on the gain curve due to the unwanted radiation along the *y*-direction. Therefore, considering all factors, selecting *l*_p_ = 3.5 mm as the final value is appropriate. 

### 2.4. Simulated Results of the Antenna Element

Based on the analysis of the proposed antenna element, one prototype of the antenna element was designed. The dimensions of the antenna element are shown as follows: *L* = 15 mm, *W* = 8 mm, *w*_p_ = 1.1 mm, *l*_p_ = 3.5 mm, *l*_s_ = 2.35 mm, *w*_s_ = 0.5 mm, *g*_s_ = 4.5 mm, *l*_a_ = 2.7 mm, *d* = 0.9 mm, *g* = 0.2 mm, *w*_m1_ = 0.68 mm, *w*_m2_ = 0.37 mm, *l*_m1_ = 1.35 mm, and *l*_m2_ = 3.34 mm.

**Figure 11:**
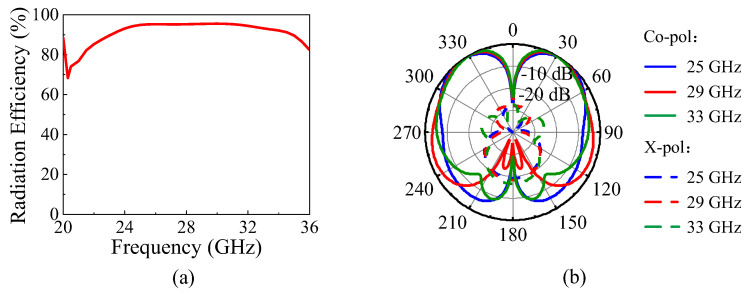
Simulated radiation efficiency and radiation patterns of the proposed antenna element. (**a**) The radiation efficiency. (**b**) The *E*-plane radiation patterns.

The simulated |*S*_11_| and the realized gain of the proposed antenna element are shown as the black curves in Figure 6. The 10 dB impedance matching bandwidth is 42.2% (22.75–35 GHz). The gain curve is flat in the operating band, and the peak gain is 5.7 dBi. Figure 11a shows the simulated radiation efficiency of the proposed antenna element, which indicates the simulated peak radiation efficiency is 95.5%. Figure 11b exhibits the simulated *E*-plane radiation patterns at 25 GHz, 29 GHz, and 33 GHz. The proposed dual-beam SIDRA has stable dual-beam radiation patterns in the *E*-plane with the maximum radiation at ±36°, ±31°, and ±30°, respectively. Their cross-polarization levels within the 3 dB beamwidth are all below −26 dB.

## 3. 1 × 4 Antenna Array and Experimental Results

### 3.1. Configuration of the Antenna Array

**Figure 12:**
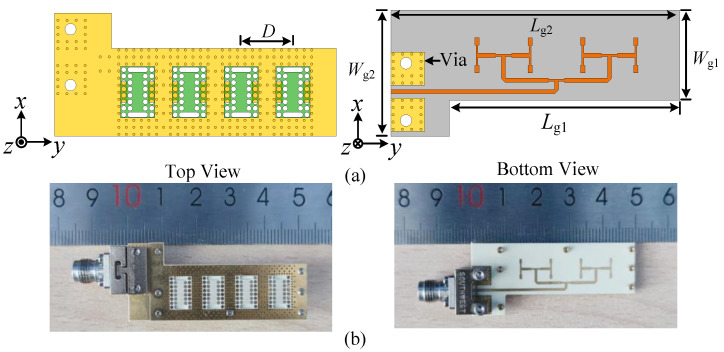
Configuration and photograph of the proposed 1 × 4 antenna array. (**a**) Configuration. (**b**) Photograph.

Based on the proposed dual-beam SIDRA element, a 1 × 4 antenna array is demonstrated. The configuration of the 1 × 4 antenna array is shown in Figure 12a. The dimensions of the antenna array are shown as follows: *L*_g1_ = 39 mm, *W*_g1_ = 15 mm, *L*_g2_ = 49 mm, *W*_g2_ = 21 mm, and *D* = 9 mm. The bottom layer is the one-to-four way feeding network. The end-launch connector is placed in the non-polarized direction of the antenna array to minimize its impact on the radiation performance of the antenna array during testing. The outline of the entire antenna array is a L-shaped structure, maintaining symmetry of the main radiation body in the *x*-direction when adding the end-launch.

### 3.2. Experimental Results

Figure 12b shows the photograph of the fabricated 1 × 4 antenna array prototype. The proposed structure mainly includes four layers of metal (Grounds 1, 2, and 3, the T-shaped feed line), three layers of substrate (Substrates 1, 2, and 3), the air vias, the air slots, and the metallic vias. In the manufacturing process, Substrate 1 is Rogers RT6010 substrate (*ε_r_*_1_ = 10.2 and loss tangent (tan*δ*_1_) = 0.0023) with a height of *h*_1_; Substrates 2 and 3 are Rogers RO4003C substrate (*ε_r_*_2_ = 3.38 and tan*δ*_2_ = 0.0027) with heights of *h*_2_ and *h*_3_. The metal layers are copper with the thicknesses of 0.017 mm (Grounds 1 and 3, the T-shaped feed line) and 0.034 mm (Ground 2). The other sizes of the structure are the same as those given in Figure 1 and Figure 12.

The *S*-parameter was measured by the Keysight N5230C vector network analyzer (Keysight Technologies, California, USA). The gain and radiation patterns were measured inside an anechoic chamber with a far-field antenna measurement system. For objective verification, the simulation was carried out with the end-launch connector. 

**Figure 13:**
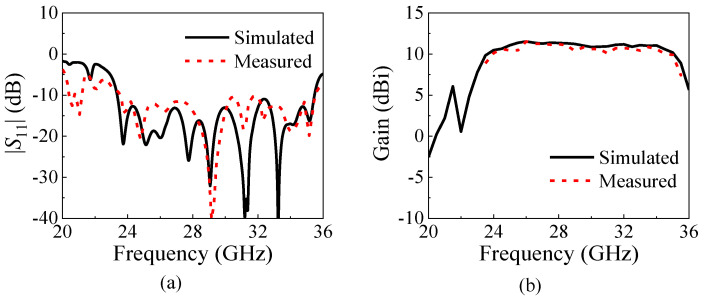
Simulated and measured |*S*_11_| and gains of the proposed 1 × 4 antenna array. (**a**) |*S*_11_|. (**b**) The gains.

The simulated and measured |*S*_11_| of the proposed 1 × 4 antenna array are shown in Figure 13a. It can be found that the measured results of the antenna array agree well with the simulated ones, and the antenna array achieves a wide 10 dB bandwidth of 41.2% (23.36–35.47 GHz), which has a slight difference in frequency and bandwidth compared with the antenna element due to the existence of the feeding network. Figure 13b exhibits the simulated and measured gain of the proposed 1 × 4 antenna array. The measured gain curve is still flat and has a peak gain of 11.54 dBi. The measured gain curve is slightly lower than the simulated one due to the testing errors.

**Figure 14:**
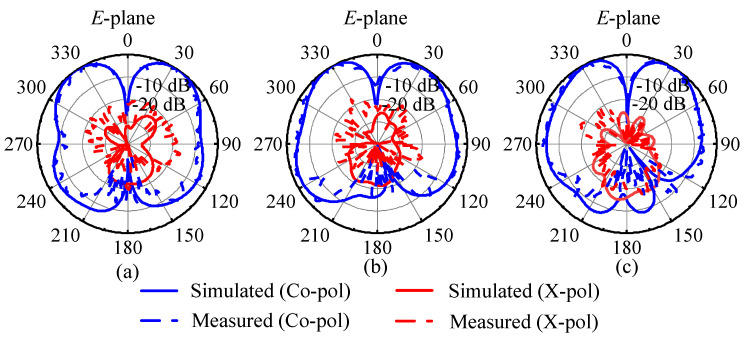
Simulated and measured *E*-plane radiation patterns of the proposed 1 × 4 antenna array at (**a**) 25 GHz, (**b**) 29 GHz, and (**c**) 33 GHz.

Figure 14 shows the simulated and measured *E*-plane radiation patterns of the proposed 1 × 4 antenna array at 25 GHz, 29 GHz, and 33 GHz, which show that the simulated radiation patterns agree well with the measured patterns. In the *E*-plane, the dual-beam radiation patterns have maximum radiation at ±35°, ±30°, and ±32°, respectively. The two beams of the *E*-plane radiation pattern are nearly symmetrical, with almost the same gain. The measured cross-polarization levels within the 3 dB beamwidth are all below −17 dB.

Comparisons between this design and the state-of-the-art antennas are summarized in Table 1. It can be seen from Table 1 that the peak radiation efficiency of the proposed array is dropped compared to the single element, which is due to the loss caused by the feed network. Compared to the metal antennas designed for dual-beam radiation with a single antenna element, this design provides a wider bandwidth and higher radiation efficiency at mm-wave band while maintaining stable dual-beam radiation. This design is more suitable for applications in the millimeter-wave applications. Compared to the existing dual-beam DRA, this design shows a wider bandwidth and a higher integration capability. Compared to the existing SIDRAs, this design represents the first attempt to achieve wideband dual-beam radiation characteristics. Also, it achieves the widest bandwidth compared with all the designs in Table 1. Therefore, the proposed dual-beam SIDRA is the preferred one considering dual-beam radiation, wide bandwidth, integration level, and mm-wave applications.

## 4. Conclusions

In this paper, a wideband dual-beam substrate integrated dielectric resonator antenna operating in mm-wave applications is proposed. In the operating band, the unwanted modes are removed by air vias and patches. Three reflection zeros provided by the dual-slot mode, the TE_211_ mode, and the TE_411_ mode help the dual-beam antenna achieve a wide bandwidth of 41.2%. For the purpose of testing, a 1 × 4 antenna array was fabricated. The overall antenna array was manufactured using substrate integrated technology, featuring a compact and planar structure with minimal assembly errors. It possesses inherent advantages when operating in mm-wave applications.

## Figures and Tables

**Table 1:** Comparison between the proposed design and the state-of-the art antennas.

Ref.	Antenna Type	*f*_0_ (GHz)	Sizes (λ_0_ × λ_0_)	10 dB FBW ^1^ (%)	Radiation Pattern	Peak Radiation Efficiency (%)
[14]	Patch	5.49	0.62 × 0.49	11.3	Dual-beam	95 (element)
[15]	Patch	29.35	1.55 × 1.47	10.6	Dual-beam	-
[16]	Patch	5.38	0.81 × 0.72	19.3	Dual-beam	~87 (element)
[17]	Patch	3.5	1 × 3.7	23.5	Dual-beam	-
[18]	DRA	6.4	0.77 × 0.77	4.7	Dual-beam	-
[21]	SIDRA	9.8	1.25 × 1.21	33.4	Broadside	92 (element)
[22]	SIDRA	24	0.64 × 0.88	34	Broadside	93 (element)
This work	SIDRA element	29	1.8 × 0.75	42.2	Dual-beam	95.5
	SIDRA array	29.4	4.8 × 2.06	41.2	Dual-beam	83

^1^ 10 dB FBW: fractional bandwidth with 10 dB impedance matching.

## Data Availability

The data presented in this study are available on request from the corresponding author.
